# Molecular Image Analysis: Quantitative Description and Classification of the Nuclear Lamina in Human Mesenchymal Stem Cells

**DOI:** 10.1155/2011/723283

**Published:** 2010-07-20

**Authors:** Christiaan H. Righolt, Vered Raz, Bart J. Vermolen, Roeland W. Dirks, Hans J. Tanke, Ian T. Young

**Affiliations:** ^1^Department of Imaging Science & Technology, Delft University of Technology, Lorentzweg 1, 2628 CJ Delft, The Netherlands; ^2^Department of Human Genetics, Leiden University Medical Center, 2300 RC Leiden, The Netherlands; ^3^Faculty of Science and Technology, University of Twente, 7500 AE Enschede, The Netherlands; ^4^Department of Molecular Cell Biology, Leiden University Medical Center, 2300 RC Leiden, The Netherlands

## Abstract

The nuclear lamina is an intermediate filament network that provides a structural framework for the cell nucleus. Changes in lamina structure are found during changes in cell fate such as cell division or cell death and are associated with human diseases. An unbiased method that quantifies changes in lamina shape can provide information on cells undergoing changes in cellular functions. We have developed an image processing methodology that finds and quantifies the 3D structure of the nuclear lamina. We show that measurements on such images can be used for cell classification and provide information concerning protein spatial localization in this structure. To demonstrate the efficacy of this method, we compared the lamina of unmanipulated human mesenchymal stem cells (hMSCs) at passage 4 to cells activated for apoptosis. A statistically significant classification was found between the two populations.

## 1. Introduction

Quantitative molecular imaging is a relatively recent research field, which is composed of two distinct domains. In one domain, the spatial resolution has a lower bound around 1 millimeter and typical methodologies are fMRI, PET, and SPECT. In the other domain, the spatial resolution is at the true molecular level and is typically described in nanometers. Typical imaging modalities include light microscopy, AFM, and electron microscopy. It is in this second domain that we are working.

 For those research questions where large numbers of image samples have to be processed in order to produce significant results, for example, in cell biology and medical diagnostics, light microscopy is usually the method of choice. Although the “bulk visualization” of biomolecules is not new—consider the measurements of DNA content made by Casperssen in the 1930s [1]—modern probe/marker technology has made it possible to make visible specific DNA sequences such as telomeric DNA and proteins such as actin and lamin A. But with this possibility to produce images at the true molecular level, the challenges increase. We no longer image volumes but rather points (gene probes), lines (actin fibers), and surfaces (nuclear lamina). While the processing of points in three-dimensional (3D) images is relatively well understood in both microscopy and astronomy, the 3D processing of lines and surfaces presents significant challenges as weak signals can destroy connectivity in lines and topology in surfaces. In this paper, we present a methodology to process one type of surface, the nuclear lamina, which is made visible through molecular imaging. The tools that we present, however, are appropriate for use in a variety of molecular imaging problems.

 In all eukaryotic cells, the nuclear envelope (NE) forms a boundary between the nucleus and the cytoplasm and thereby physically separates nuclear from cytoplasmic activities. The NE consists of an outer nuclear membrane (ONM) fused through nuclear pore complexes with an inner nuclear membrane (INM), which is underlined by the nuclear lamina [2]. The nuclear lamina, which is on the order of 30–100 nm thick [3], is primarily composed of B-lamin proteins and the lamin A/C-types. The lamin B proteins are constitutively expressed and essential for the organism; lamin B knock-down mice are nonviable and die at birth [4]. The lamin A/C gene is developmentally regulated and mutations in the lamin A gene cause a broad spectrum of hereditable human diseases which are collectively called laminopathies (reviewed in [5]). The nuclear lamina provides a structural framework for the cell nucleus and high resolution imaging techniques reveal its structure. The nuclear lamina is composed of a fibrous network of lamin filaments together with membrane-associated proteins and was first identified in vertebrates by electron microscopy [6]. It was recently resolved using cryo-electron tomography [7]. The nuclear lamina is a highly dynamic structure and changes in nuclear lamina structure are associated with many cellular processes such as cell division, cell differentiation, cell senescence and apoptosis (reviewed in [8]). In addition, lamin proteins are involved in the regulation of nuclear functions such as transcription, replication, and DNA repair and they can directly bind both euchromatic and heterochromatic regions [9–11]. Cells expressing mutations in the lamin gene exhibit a deformed nuclear shape which is associated with changes in transcriptome, DNA damage and DNA methylation [12]. It has, therefore, been proposed that the nuclear lamina can affect the spatial positioning of nuclear structures which subsequently affect nuclear functions. How the nuclear lamina changes its shape, however, is still not clear. By studying the 3D structure of the lamina, we can, therefore, expect to see the spoors of changes in cellular processes. 

 The lamin proteins are direct targets of cell-death-activated caspases [13]. Upon activation of apoptosis, the lamin proteins are cleaved by the apoptosis-activated caspases and followed by DNA fragmentation, the hallmark of apoptosis [13]. Previous studies indicate that, during activation of caspase-8 in hMSCs, changes in lamina spatial organization, including invagination of the lamina into the nuclear sphere and the formation of intranuclear lamina structures, can be visualized before cleavage of lamin B by caspase-3 and breakdown of the nuclear lamina [14].

**Figure 1:**
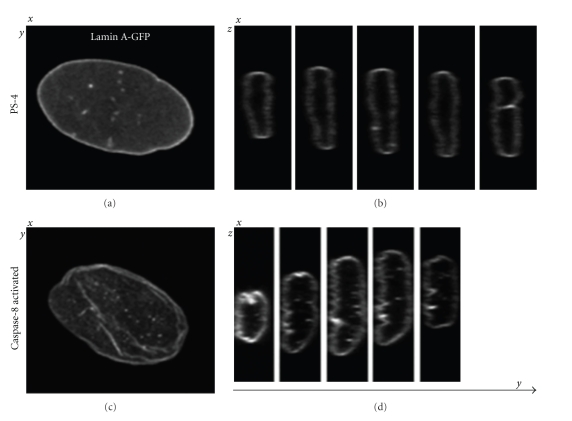
Confocal images of lamin A-GFP in hMSCs. The hMSCs at passage 3 were transduced with the lamin A-EGFP lentiviral vector and after 3 days 1/2 of the cells were cotransduced with FKC8 lentivirus vector. For caspase-8 activation, cells were treated with 100 nM AP20187 for 4 hours. Confocal Z-stacks (*z* = 122 nm) were taken from living cells. The *x*-*y* images of 35 × 35 *μ*m^2^ show a maximum projection, and the sequential *x*-*z* image sections show equal intervals along the *y*-axis. Top images are of a typical passage 4 cell (PS 4), and bottom images are of a typical caspase-8 activated (FKC8) cell.

 The intranuclear lamina structures can be recognized in vertical, optical sections of cells as shown in Figure 1. It is not yet clear how these intranuclear structures are formed or what their functions are but there is a spatiotemporal correlation with the occurrence of telomere aggregates [11]. While yeast does not contain lamins, telomere aggregates have been observed and are associated with gene silencing [15]. It is possible, therefore, that lamina intranuclear structures play a regulatory role in nuclear function.

 To comprehensively study the spatial changes in nuclear lamina in cellular processes, unbiased quantitative description of the lamina structure should be applied. So far, a quantitative description of this structure has not yet been provided. We have developed an imaging method that segments the nuclear lamina resulting in a quantitative description of this structure using two key steps, segmentation and measurement, from which unbiased quantitative spatial information obtained from the lamina structure can be generated and statistically evaluated.

 To demonstrate the biological relevance of this method, we have extracted lamina features from un-manipulated and caspase-8 activated cell populations and used these as a basis for classification. Based on these lamina features, a linear separation was found between the two-cell populations. Altogether our results demonstrate that changes in lamina structure are measurable and can be used as a tool to objectively distinguish healthy from unhealthy cells. Moreover the method can be used to understand how the shape of the nuclear lamina relates to its biological function. We suggest that biophysical features of the nuclear lamina can be used as a research tool to associate changes in lamina structure with cellular processes.

## 2. Materials and Methods

### 2.1. Simulation Studies

The techniques used in this quantitative analysis have been developed over a number of years and reported in a number of publications. In particular, simulation studies have been performed on 3D image restoration [16, 17], the measurement of curvature [18, 19] and the segmentation of objects in the presence of noise [20]. For the work described here, additional simulation studies involved the accurate measurement of the curvature associated with the thin lamina membrane. Space considerations do not permit discussing the details of these experiments but the details of this extensive work can be found in [21].

### 2.2. Biological Material

To visualize the nuclear lamina in living cells, we expressed the green fluorescent protein (GFP) fused to the lamin A gene in human mesenchymal stem cells (hMSCs) using a lentivirus expression system [14]. Upon transduction, the lentiviral DNA, containing lamina-GFP, is stably integrated into the host genome allowing repeated imaging during long-term culture. As only one or two copies are integrated into the host genome, high overexpression of the transgene is generally precluded. To avoid transgene overexpression artifacts, cells with high fluorescence intensity were excluded from these studies. 

 Human MSCs were isolated from bone marrow samples obtained as described in [22]. Cells were propagated in vitro as described in [14]. The lentiviral vectors used in this work are the so-called self-inactivating (SIN) vectors [23]. The fusion genes FKC8 (inducible caspase-8) and lamin A-EGFP were described previously [14, 24]. The viral production and the hMSCs virus transduction procedures were carried out as described previously [14]. After transduction cells were cultured for additional passages without any selection pressure and without losing the transduced genes. The protein expression pattern observed after 8-9 passages was similar to the expression pattern of the endogenous proteins as verified with immunocytochemistry. Activation of the inducible caspase-8, FKC8, in the hMSCs was carried out with 100 nM AP20187 (ARIAD), as described in [14].

### 2.3. Immunofluorescence

Immunofluorescence of cells seeded on glass plates and fixed prior to antibody incubation was carried out as previously described [14]. The mouse-antihuman lamin A antibody (1 : 1000; Santa Cruz) was used to detect lamin A protein.

### 2.4. Microscopy and Image Processing

Image stacks (Δ*z* = 122 nm) were collected from living cells using a confocal microscope and lamina spatial structure was defined after 3D reconstructions (Figure 1). The nuclear lamina, in cells at passage 4, showed a smooth ellipsoid-like shape (Figure 1, PS 4). After caspase-8 activation, however, the lamina structure was distorted (Figure 1, Caspase-8 activated). These images were further used for the development of a quantitative description of the nuclear lamina. 

 Images were recorded with a Leica TCS SP2 confocal microscope using an oil-immersion objective with a magnification of *M* = 63× and a numerical aperture of NA = 1.32. In this imaging system the point spread function (psf) is anisotropic for the axial and lateral directions. This means that the amount of spatial blurring will differ between these orthogonal directions and, due to the differing Nyquist criteria, the voxel size can differ in each direction without loss of information. The voxel sizes were 162.8 nm in the axial direction and in the lateral direction 52.6 nm for the passage 4 cells and 69.3 nm for the caspase-8 activated cells. The differences in voxel size in the lateral direction for the cell types reflect different scanning settings. The measurements that we use were normalized to be independent of these voxel sizes. The three-dimensional images are processed in DipImage, a software package developed at the Delft University of Technology (http://www.diplib.org/), running under the MatLab environment (The MathWorks, Natick, Massachusetts). 

 As the lamina thickness is quite small < 20% of the wavelength of emission light (*λ* ≈ 500 nm), the laminar image is blurred by the point spread function (psf) of a confocal imaging system [25]. This blurring however, does not mask the spatial changes that occur in lamina morphology (Figure 1). For a quantitative description of the lamina structure, two key steps were applied: segmentation and measurement. 

### 2.5. Segmentation

Segmenting the lamina means finding the thin membrane that is so evident in Figure 1. The segmentation step is used to determine which of the three-dimensional voxels are associated with the nuclear lamina. The first step requires the creation of isotropic images from the recorded anisotropic images. Isotropic images are required because the measurements are voxel based so equal voxel sizes are, in general, required if the measurements are to be independent of cell orientation. The two types of anisotropic behavior in these images are (1) the difference between axial and lateral voxel size and (2) the difference in the amount of axial and lateral blurring. Both of these effects are inherent in confocal microscopy. Methods to eliminate both of these anisotropies are presented here. 

 In the first step images were resampled such that the voxels have equal lengths in all directions. The lateral voxel sizes (*x* and *y* directions) are approximately 60 nm and the axial size (*z* direction) has to be resampled from 160 nm to 60 nm. This was accomplished by linear interpolation of pixel values [26]. 

 The next step was correction for the anisotropy of the blurring induced by the anisotropic psf of a confocal microscope. We assumed a diffraction-limited, aberration-free objective lens. Because of the circular symmetry of the three-dimensional psf in the lateral plane, it can be described by an axial coordinate *z* and a radial coordinate *r*. With a minimum square error fit, a Gaussian function can be shown to be an excellent model for this 3D psf with two width parameters *σ*_*r*_ (lateral) and *σ*_*z*_ (axial) [27].

 Because we require isotropic images, the amount of blurring must be equal in each direction *r* and *z*. As *σ*_*r*_ < *σ*_*z*_, the image has to be blurred in the radial direction with a Gaussian of size

(1) $$\sigma_{r,\text{blur}}^{2}=\sigma_{z}^{2}-\sigma_{r}^{2}$$

as variances are additive in a Gaussian function. Using the values from our Gaussian fit to the psf this leads to *σ*_*r*_ = 62 nm, *σ*_*z*_ = 190 nm and thus *σ*_*r*,blur_ = 180 nm.

**Figure 2:**
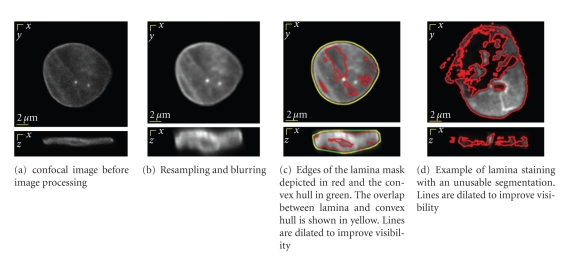
Result of lamina image processing for cells at passage 4: (a,b) anisotropy is caused by spatial sampling and an anisotropic objective lens and (c) lamina segmentation. Top: *x*-*y* slice; Bottom: *x*-*z* slice. (a–c) image from a living cell, lamin A is visualized with a GFP fusion; (d) an image from a fixed cell, lamin A is detected with antibody staining.

Slices from the resampled and blurred, now-isotropic images are shown in Figures 2(a) and 2(b). At this point and in subsequent processing the images intensities are in a real number (floating point) representation and are no longer treated as integer values.

The key to segmentation is a variation on the theme of “unsharp masking” [28]. In unsharp masking we emphasize edges by subtracting a smoothed version of an image from the original image. The unsharp masking method works as follows

 For the 3D image *i*(*x*, *y*, *z*) we compute:

(2) $$\begin{array}{cc} & i_{\text{unsharp-masking}}\left(x,y,z\right)\\ & \;\;=i_{\text{isotropic}}\left(x,y,z\right)-\left(\alpha \cdot i_{\text{blur}}\left(x,y,z\right)+\beta \right), \end{array}$$

where *α* and *β* are constants. The voxels associated with the lamina are now found by thresholding the unsharp-masked image. This means that we are using a form of local thresholding to determine the lamina voxels and the determination of *α* and *β* is described below

(3) $$i_{\text{mask}}\left(x,y,z\right)=\{\begin{array}{cc} 1, & i_{\text{unsharp-masking}}\left(x,y,z\right)\geq 0,\\ 0, & i_{\text{unsharp-masking}}\left(x,y,z\right)<0. \end{array}$$

The assignment of *i*_mask_ for the case when *i*_unsharp-masking_ = 0 is not critical as we are using a real number representation. Since the local threshold depends on the signal-to-noise ratio (SNR) in the neighborhood of a voxel, the lower intensity parts of the lamina are more difficult to segment. The SNR can, in fact, be caused by a number of sources: dark current, photomultiplier tube noise, electronics noise and photon noise. As discussed in the literature [29, 30] and as our experiments deal with small, weakly-emitting structures, the dominant source of noise in our images is photon (Poisson) noise and the SNR is proportional to $\sqrt{\langle i(x,y,z)\rangle }$ where 〈*i*(*x*, *y*, *z*)〉 is the average intensity associated with the Poisson process that led to that intensity value.

 The image *i*_blur_ in (2) is a Gaussian-smoothed version of *i*_isotropic_:

(4) $$\begin{array}{cc} i_{\text{blur}}\left(x,y,z\right) & =G_{\sigma_{L},\sigma_{A}}\left(x,y,z\right)⊗i_{\text{isotropic}}\left(x,y,z\right)\\ & =G_{\sigma_{L}}\left(x,y\right)⊗\left(G_{\sigma_{A}}\left(z\right)⊗i_{\text{isotropic}}\left(x,y,z\right)\right)\\ & =G_{\sigma_{L}}\left(r\right)⊗\left(G_{\sigma_{A}}\left(z\right)⊗i_{\text{isotropic}}\left(x,y,z\right)\right). \end{array}$$

Importantly, (4) indicates that we make use of the separability of the Gaussian in the lateral and axial directions and the fact that the Gaussian is circularly symmetric in the lateral plane. The values of the parameters *σ*_*L*_ and *σ*_*A*_ are determined as follows. The lateral smoothing parameter (*σ*_*L*_) is based upon the lateral extent of the lens psf. The total lateral psf extent, using the Abbe half-width criteria of 0.5*λ*/NA, is given by (*λ* = 509 nm)/(NA = 1.32) = 385 nm. Our sampling density in the lateral direction is about 60 nm/voxel. We use *σ*_*L*_ = 1 voxel = 60 nm ≈ 1/6 of the psf lateral extent so as to provide smoothing of the image without excessive blurring of the thin lamina image. We note that the additional smoothing realized by this step is marginal compared to the smoothing that has taken place above, where *σ*_*r*,blur_ = 180 nm. This particular step, in fact, increases the effective total filter *σ* in the lateral direction from 180 nm to 190 nm. (See (1).) Combining the two filter steps in one step would certainly increase the computational speed. We describe this, however, as two steps in order to indicate where the various contributions that require filtering originate.

**Figure 3:**
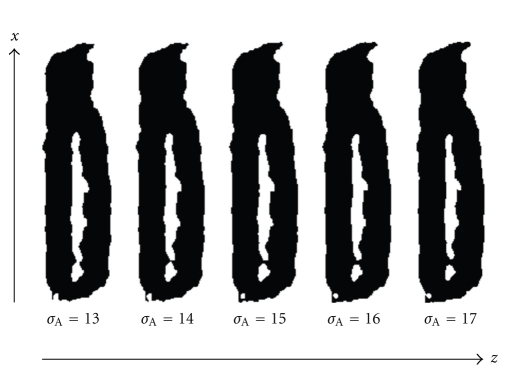
Segmentation results for the nonspherical lamina for varying values of the axial smoothing parameter ranging from *σ*_*A*_ = 13 to *σ*_*A*_ = 17. The segmentation is essentially constant over this 15% variation. The value *σ*_*A*_ = 15 is used in this work.

 The axial smoothing parameter (*σ*_*A*_) is similarly chosen. The total axial psf extent [31] is given by 4*λ*/NA^2^ = 4 · 509 nm/(1.32^2^) = 1169 nm. We use *σ*_*A*_ = 15 voxels with an axial resampled density of 60 nm which gives an extent of 900 nm ≈ 3/4 of the psf axial extent. The stability of our segmentation result for changes in *σ*_*A*_ is illustrated in Figure 3. A 15% change in *σ*_*A*_ has no significant effect on the result. This also holds for *σ*_*L*_ (data not shown).

**Figure 4:**
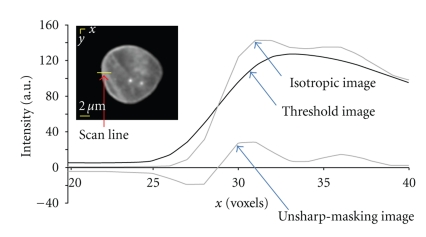
An illustration of the unsharp-masking procedure where a section is scanned along one line and the isotropic, threshold, and unsharp-masking “images” are calculated for this single scan line. The procedure is actually performed in three dimensions.

 This mask now depends on the two parameters (*α*, *β*) in (2). The intensity differences across the lamina do not allow a global threshold, so a local thresholding technique is used. As shown in Figure 4, the image *i*_threshold_(*x*, *y*, *z*) = *α* · *i*_blur_(*x*, *y*, *z*) + *β* has higher values of intensity for the background than *i*_isotropic_(*x*, *y*, *z*) and lower values inside the cell than *i*_isotropic_(*x*, *y*, *z*). This can be accomplished by choosing 0 < *α* < 1 and *β* > 0. At those positions where we made the transition from inside to outside the lamina, we require that *i*_unsharp-masking_ = 0. Solving for the value of intensity *I*_*c*_ where this transition occurs gives *I*_*c*_ = *β*/(1 − *α*). 

 We began by setting *I*_*c*_ at a somewhat lower value than the average of max [*i*_blur_] and min [*i*_blur_]. The reason is that, for Poisson noise, the noise level at higher intensities is higher *on an absolute scale* than at low intensities. We choose, therefore, for *I*_*c*_ = max [*i*_blur_]/3. In order for the unsharp mask to accurately follow the shape of *i*_isotropic_ as seen in Figure 4, we set *α* = 0.9. This choice means that *β* = max [*i*_blur_]/30. The resulting unsharp-masking image, defined in (2) and used in (3), is also shown in Figure 4. Again, the segmentation results are not overly sensitive to our chosen values *α* and *β*, data not shown. 

 Thresholding schemes in 2D images produce contours and in 3D they produce surfaces. In both cases gaps can appear in what should be the closed contour or surface. The holes that may appear can essentially be eliminated by using a morphological closing [28]. A closing is a dilation followed by an erosion on the mask image computed in (3). Care must be taken in choosing the size of the closing, the structuring element, as it must be sufficiently large to close the holes but small enough to leave the intranuclear space open. We used a digital approximation to a sphere of radius 2.7 voxels (162 nm), which contains 81 voxels. The structuring element is isotropic because the image is isotropic even though the psf is not. Segmentation results are shown in Figure 2(c).

 All of the 49 cells used in this study, that is 100%, produced successful segmentation results that were suitable for further processing. In comparison, when the nuclear lamina was marked with antibodies against lamin A on fixed cells, only 30 out of 42 cells (71%) produced segmentation results that were suitable for further processing. An example of an unusable segmentation result from a fixed cell is shown in Figure 2(d). These results show that the lamina segmentation method can be applied also on cells, where the lamina is visualized with immunolabeling. However due to the significant number of unusable segmentation results it is more laborious. Further, the use of fixed cells does not afford the flexibility associated with live cell studies. In the study we used segmentation results that were obtained from living cells. 

### 2.6. Measurement

For a quantitative description of nuclear lamina shape, three features were measured from the segmented images: the average normalized intensity, skewness of the intensity distribution, and normalized average absolute Gaussian curvature. These features were initially suggested by visual inspection of the nuclear lamina. Here we converted features visualization into mathematical descriptors.

 The intensity-based measures begin with the intensities of those voxels that are located in the lamina mask produced by the segmentation procedure described above. The mean (*μ*) and standard deviation (*σ*) of this set of intensities were collected and outliers—values that are more than 4*σ* from *μ*—were clipped to either *μ* − 4*σ* or *μ* + 4*σ*. The intensities were then normalized to the interval (0,1) using

(5) $$i_{\mathrm{norm}\;}=\frac{i-\min\;\left(i\right)}{\max\;\left(i\right)-\min\;\left(i\right)}.$$

The average normalized intensity of the laminar voxels is computed as follows:

(6) $$i_{\text{average}}=\frac{\sum_{\text{mask}}i_{\text{norm}}}{\sum_{\text{mask}}}.$$

The skewness of the intensity of the laminar voxels is computed as follows:

(7) $$i_{\text{skewness}}=\frac{\sum_{\text{mask}}{\left(\left(i_{\mathrm{norm}\;}-i_{\text{average}}\right)/\sigma_{i}\right)}^{3}}{\sum_{\text{mask}}},$$

where *i*_average_ is defined in (6) and *σ*_*i*_ is the standard deviation of the normalized intensity distribution. Both *i*_average_ and *σ*_*i*_ can be calculated from a histogram of the intensity values associated with (5). The denominator of (6) and (7) is the number of voxels associated with the lamina and it guarantees that this measure is independent of voxel size.

**Figure 5:**
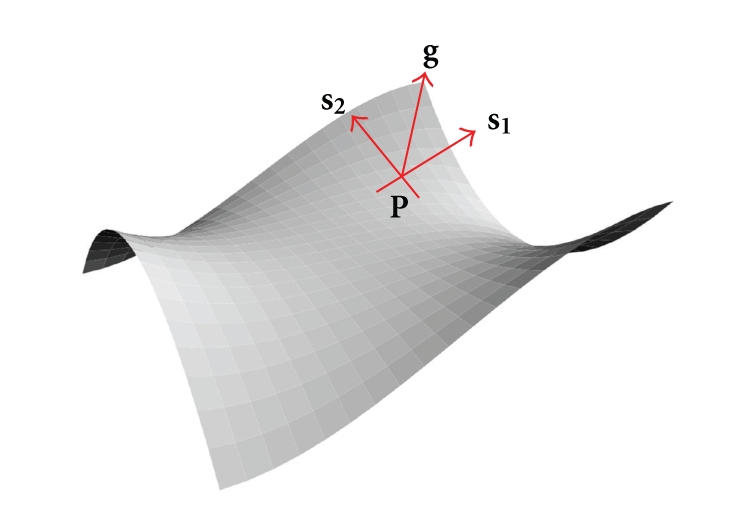
The surface has at every point **P**, a gradient **g** normal to the surface and two, orthogonal, surface vectors, **s_1_** and **s_2_**. The two vectors are orthogonal to **g **and to one another.

 The curvature measure is defined by the voxel mask resulting from segmentation, and it describes the rate at which the direction of a path changes along the lamina surface (Figure 5). The concepts “rate” and “direction” both require the use of a derivative so calculation of curvature involves both first and second derivatives. We use the two principal curvatures (*κ*_1_ and *κ*_2_) [32] and these two curvatures are combined to yield the Gaussian curvature *K* = *κ*_1_ · *κ*_2_ at every point on the laminar surface. These curvatures provide a mathematical description of the way the surface bends.

Calculation of the curvature of a (laminar) surface in a three-dimensional space is straightforward and is described below. At any point **P** on the lamina surface there is a gradient vector **g** that is normal to the surface and two additional surface vectors, **s_1_** and **s_2_**, that (1) lie on the surface, (2) are orthogonal to the normal vector and (3) orthogonal to one another. This can be illustrated by a “monkey saddle” (See http://en.wikipedia.org/wiki/Monkey_saddle/) as shown in Figure 5.

 Each choice of **s_1_** corresponds to a path going through **P** and a curvature of that path at **P**, *κ* **(P)**. As we rotate **s_1_** around **g**, *κ*_1_ and *κ*_2_ are the maximum and minimum curvatures that go through the point **P**. A positive curvature corresponds to a convex bending, a negative curvature to a concave bending, and a zero curvature to no bending. The product of these two curvatures is the Gaussian curvature. These calculations have to be performed properly and procedures are described in [32, chapters 16 and 17] and in [33].

Two parameters must be chosen to ensure that the curvature-associated derivatives are correctly calculated: the size *σ*_*g*_ for a Gaussian derivative filter used to implement the derivatives and the size *σ*_*w*_ of an averaging window for the gradient structure tensor (GST) [18, 34]. It is important that the measurement results are stable for small deviations in *σ*_*g*_ and *σ*_*w*_. This stability of the results means that the curvatures can be meaningfully compared between different images. We have determined that, given the size of our voxels, *σ*_*g*_ = 3 voxels (≈180 nm) and *σ*_*w*_ = 7 voxels (≈420 nm) lead to stable results, data not shown. These values for *σ*_*g*_ and *σ*_*w*_ are consistent with the values reported in [18, 33].

The normalized, average, absolute Gaussian curvature is then computed as

(8) $$K_{\text{naaGc}}=A_{\text{CH}}\left(\frac{\sum_{\text{mask}}\left|K\right|}{\sum_{\text{mask}}}\right).$$

The term within the parentheses is the average, absolute Gaussian curvature. Its dimensions are length^−2^. The normalization term, *A*_CH_, is the area of the convex hull that encompasses the laminar surface. The convex hull of the mask is taken in order to neglect the internal structure and to find a closed object with dimensions comparable to the dimensions of the mask. A two dimensional example of this can be seen in Figure 2(c). The convex hull term ensures that the curvature measure is independent of voxel size.

### 2.7. Data Analysis

We use simple, linear classifiers based upon the Fisher linear discriminant [35] to show that these measurements are sufficient to distinguish between passage 4 and caspase-8 activated cells. The Fisher discriminant allows determination of the decision lines shown in Figure 7 and subsequent classification resulting in the confusion matrix presented in Table 1. These statistical and classification procedures are embodied in PRTools, a software package for MatLab developed at the Delft University of Technology (http://www.prtools.org/).

## 3. Results

### 3.1. Spatial Localization

**Figure 6:**
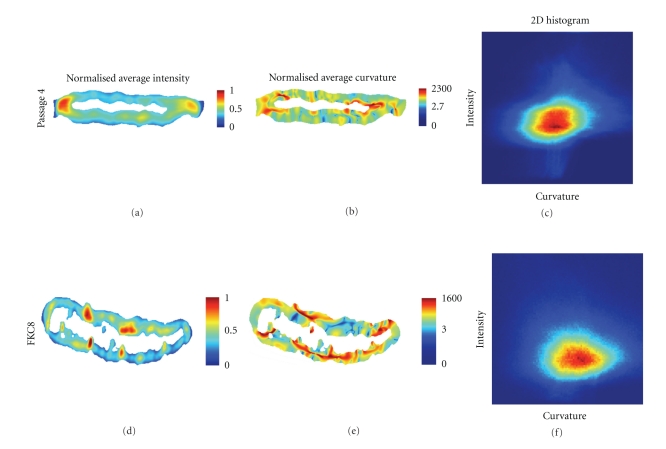
Local, spatial distribution of measurements in a single, 2D (*x*, *z*) slice of a lamina for a representative cell at passage 4 and for another representative cell after caspase-8 (FKC8) activation. (a) and (d): Intensity values with a *linear* scale; (b) and (e): Curvature values with a *logarithmic* scale; (c) and (f): The 2D histogram shows values of intensity and curvature pairs for each and every voxel of the 3D lamina structure.

The intensity and curvature measures were used to give a local spatial distribution of intensity (6) and curvature (8) in the lamina structure of a specific hMSC. To produce the image of a typical cell shown in Figure 6, the summed and normalized values for intensity or curvature over an entire 3D lamina were projected onto a single cross section per cell.

 For the normal cells used in this study, high intensity, which indicates accumulation of lamin A, is found at the left and right edge of two-dimensional slices of the three-dimensional (nonspherical) nucleus (Figures 6(a), 6(c), and 6(f)). The high curvature regions do not, however, show spatial correlation with the intensity as demonstrated by the 2D histogram and the low correlation coefficient *ρ* = 0.265 (Figures 6(a), 6(b), 6(e), 6(c), and 6(f)). This histogram was computed by plotting the intensity versus curvature for each voxel in the lamina and the correlation coefficient was extracted from this histogram. The number of voxels involved in calculating this correlation coefficient was 4,607,349.

 Since the structure of the nuclear lamina is dramatically changed during apoptosis activation [11, 12], we have compared the spatial and structural changes of the nuclear lamina in un-manipulated cells to those that were activated with the inducible caspase-8 (FKC8). Activation of hMSCs with FKC8 leads to cell death which is characterized by cleavage of caspase-3, lamin B and lamin A proteins and DNA fragmentation, as detected by the TUNNEL assay [12].

 Here we found that in FKC8-activated cells, the distribution of lamin A-GFP differs, as compared to the untreated cells. After FKC8 treatment high intensity and high curvature were found at the upper and lower surfaces of the nucleus indicating redistribution and local accumulation of lamin A proteins. (Figure 6(f) shows a typical caspase-8 activated cell.) The intensity and curvature correlation found after caspase-8 activation was *ρ* = −0.057 indicating that these two features were essentially uncorrelated. The number of voxels involved in calculating this correlation coefficient was 2,226,379.

 In these two-cells, which are typical of the cells that we analyzed in this study, the high intensity regions are surrounded by high curvature values. In the FKC8-activated cells, however, additional high-curvature regions, which are not associated with local accumulation of lamin A, are also detected. These results suggest that bending of the nuclear lamina can result from local accumulation of lamin proteins but additional factors, such as lamina-associated proteins, can also affect the bending of this structure. (The relationship between bending and curvature is described in [33, 36].)

### 3.2. Cell Classification

In order to systematically analyze the changes in lamina organization, we measured lamina features from un-manipulated (control) cells at passage 4 and compared them to caspase-8 activated cells. We chose the caspase-8 activated cells to model our quantitative lamina description method as changes in lamina organization are one of the initial characteristics found during activation of caspase-8 [14].

**Figure 7:**
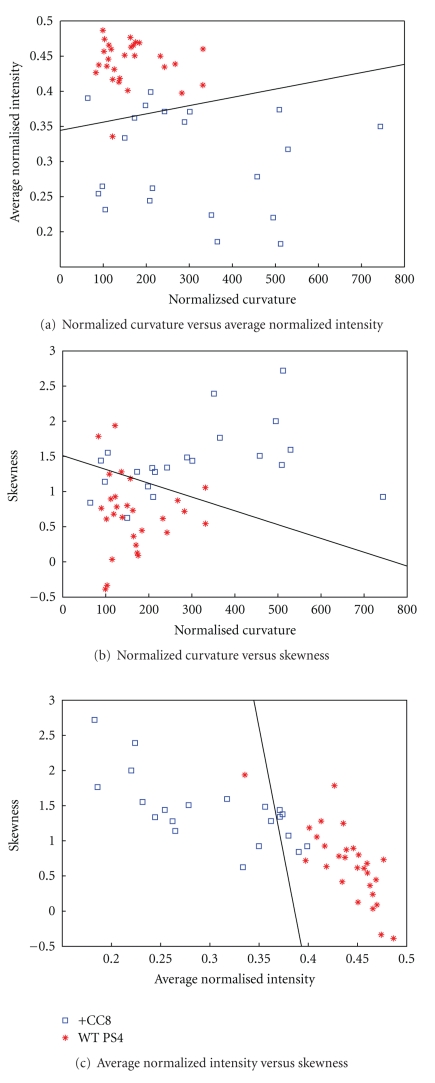
Scatter plots and linear classification lines of passage 4 (PS 4) versus caspase-8 activated cells are shown for each two-feature combination. The number of cells per datasets is: *N*_PS4_ = 28, *N*_caspase-8  activated_ = 25. Passage 4 cells (WT PS4) are shown with (*) and caspase-8 cells (+CC8) with (□).

 The values of mean intensity, skewness and mean curvature were measured for every cell, plotted in 2D scatter graphs (Figure 7), and subsequently used in classification tests. “Classical” statistical classification procedures were used to distinguish between the two populations. More recent techniques, such as support vector machines (SVM), were not warranted for this application given our sample size, number of features, and the data simplicity. Figure 7 illustrates the results of a linear classifier where cells on one side of the line are classified as belonging to one of the populations and cells on the other side of the line are classified as belong to the other. The data indicate that cells at passage 4 can be distinguished from caspase-8 activated cells on the basis of every two-feature combination.

 The significance of the classification is demonstrated in a confusion matrix that indicates the frequency with which cell type *i* is (mis)identified as cell type *j* (Table 1). Using the confidence limits for the binomial distribution [37], these results are statistically significant (compared to the one-sided, null hypothesis that classifications are made at random) with *p*_control_ ≤ 1.4 × 10^−5^ and *p*_FKC8_ ≤ 1.3 × 10^−2^. 

 We also tested whether a quadratic classifier or the use of all three features in the classification, instead of just two, might lead to an improved performance. As shown in Table 1, no significant improvement over the two-feature, linear classifier was found. 

## 4. Discussion

We have developed an unbiased method to describe the nuclear lamina with mathematical descriptors. We show that the three features that we have chosen are sufficient to provide excellent discrimination between passage-4, un-manipulated cells and caspase-8 activated cells. This means that these features, combined in a measurement tool, can be used to follow changes in nuclear lamina morphology as a cell progresses from healthy to cell death. With this tool it should be possible to infer the viability status of the cell from the shape of its nuclear lamina. 

 The preprocessing, segmentation and measurement procedures involve the determination of values, sometimes referred to as “magic numbers”, for the six parameters (*σ*_*L*_, *σ*_*A*_, *α*, *β*, *σ*_*g*_, *σ*_*w*_). The values we have used are, to a certain extent, determined by physical parameters such as *λ*, NA, psf, SNR, and sampling distance. We have examined the sensitivity of our measurements to variations in these parameters and, as described in the methods section, small variations (≈15%) in these parameter values do not produce significant changes in the results. The parameter values, however, are also problem-dependent and selected for the specific biological model that is being studied and the biological probes and markers that are being used. That is to say, we have determined values that work well with a nuclear lamina probe. Other subcellular compartments might require adjustments.

 As indicated by the high percentage of usable segmentation results, the method we developed is suitable for living cells, where the nuclear lamina is visualized with a fluorescent protein. In addition, the segmentation is adjusted for the elliptical nuclear shape found in hMSCs. It will be important to test this method on other cells, which will allow broadening the application domain. Parameters that describe the nuclear shape may require adjustments [38]. 

 Previous studies by Rohde et al. [39] described an algorithmic tool that elegantly represents the 2D contour of the cell nucleus. Schermelleh et al. have shown how three-dimensional, structured illumination microscopy (3D-SIM) could provide images with detailed spatial information about the lamina network [40]. Neither study, however, provided a quantitative description of the nuclear lamina. To our knowledge we are the first to provide such a description and its subsequent application.

 Importantly we have introduced a visualization tool that provides spatial information concerning the nuclear lamina and shows *where* in the lamina structure morphological changes occur when cells undergo apoptosis. This tool can be used to understand how the distribution of the lamina proteins defines this structure. We have recently shown that specific nuclear elements change their spatial localization with respect to the nuclear space and nuclear lamina [11, 41]. This tool, when combined with additional nuclear probes for chromatin regions or lamina-binding proteins, should be useful in localizing the association of specific nuclear probes with the lamina. As this study is carried out in living cells it can provide additional information as to how the lamina is involved in spatial and temporal regulation of nuclear function.

 Cell death is an essential biological process for eliminating unwanted cells during development, growth, differentiation, and maintenance of tissue homeostasis. Failure to eliminate such unwanted cells may contribute to the development of pathologies. The capacity to evade (apoptotic) cell death has been defined as one of the hallmarks of cancer [42]. As a change in lamina morphology is the initial event that can be observed after activation of apoptosis [13, 14], there is a rationale to extract quantitative features of lamina morphology and to use these features to identify cells in a population that is undergoing apoptosis. Here we have developed an imaging methodology to quantify structural information, in this case the intensity and curvature of the nuclear lamina. We have demonstrated that this method allows one to accurately distinguish apoptotic cells from normal cells. Previously we have shown that the unbiased support vector machine-learning techniques can also be applied to classify between untreated and capsase-8 activated cells using images of the nuclear lamina [43]. The SVM method, although unbiased, does not provide a quantitative description of this structure.

 Recent developments in stem-cell research for application in regenerative medicine, in particular induced pluripotent stem cells (iPS), require unbiased methods for stem cell characterization. Unbiased methods, such as the one presented here, can be important in characterizing and sorting stem cells during in vitro propagation and prior to transplantation. In addition, it has been recently suggested that observing cells at early stages of apoptosis can be used to evaluate treatment efficacy or even to predict tumor responsiveness to treatment [44]. An image analysis tool that automatically identifies cells that are targeted for apoptosis can therefore be of potential use in the clinic. Here we have applied this new method on hMSCs, as the nuclear shape of healthy cells at early passage number is relatively uniform [41] even in a heterogeneous cell population such as hMSCs. As distorted nuclear shape during apoptosis has been observed in other cell types, it will be particularly interesting to apply this tool in vivo in order to mark different apoptotic cells and to evaluate the rate of cell apoptosis.

 Finally, as changes in lamina structure are found in additional biological processes, such as cells undergoing senescence or cells carrying mutations in lamina genes [5], the image analysis methodology described here might also be used to evaluate how changes in lamina structure are related to other changes in cell fate.

## Figures and Tables

**Table 1:** Confusion matrix for classification of the two-cell populations using two-feature classifications and three-feature classifications. The two-feature classifier uses normalized curvature and average normalized intensity (Figure 7(a)) with a linear classifier and a quadratic classifier. The three-feature classifier uses normalized curvature, average normalized intensity and skewness with a linear classifier and a quadratic classifier. The percentage classification is given together with the standard error of its estimate (*μ* ± *σ*).

		Biological sample	
		Passage 4 cells	FKC8 cells
		*N* = 28, 100%	*N* = 21, 100%
Statistical classification (2 features)	Linear classifier		
	passage 4	27, 96% ± 4%	3, 14% ± 8%
	FKC8	1, 4% ± 4%	18, 86% ± 8%
	Quadratic classifier		
	passage 4	26, 93% ± 5%	3, 14% ± 8%
	FKC8	2, 7% ± 5%	18, 86% ± 8%

Statistical classification (3 features)	Linear classifier		
	passage 4	27, 96% ± 4%	5, 24% ± 9%
	FKC8	1, 4% ± 4%	16, 76% ± 9%
	Quadratic classifier		
	passage 4	25, 89% ± 6%	1, 5% ± 5%
	FKC8	3, 11% ± 6%	20, 95% ± 5%
